# DNA Methylation of T1R1 Gene in the Vegetarian Adaptation of Grass Carp *Ctenopharyngodon idella*

**DOI:** 10.1038/s41598-018-25121-4

**Published:** 2018-05-02

**Authors:** Wenjing Cai, Shan He, Xu-Fang Liang, Xiaochen Yuan

**Affiliations:** 1 College of Fisheries, Chinese Perch Research Center, Huazhong Agricultural University, Wuhan, 430070 China; 2 Freshwater Aquaculture Collaborative Innovation Center of Hubei Province, Key Lab of Freshwater Animal Breeding, Ministry of Agriculture, Wuhan, 430070 China

## Abstract

Although previous studies have indicated importance of taste receptors in food habits formation in mammals, little is known about those in fish. Grass carp is an excellent model for studying vegetarian adaptation, as it shows food habit transition from carnivore to herbivore. In the present study, pseudogenization or frameshift mutations of the umami receptors that hypothesized related to dietary switch in vertebrates, were not found in grass carp, suggesting other mechanisms for vegetarian adaptation in grass carp. T1R1 and T1R3 strongly responded to L-Arg and L-Lys, differing from those of zebrafish and medaka, contributing to high species specificity in amino acid preferences and diet selection of grass carp. After food habit transition of grass carp, DNA methylation levels were higher in CPG1 and CPG3 islands of upstream control region of T1R1 gene. Luciferase activity assay of upstream regulatory region of T1R1 (−2500-0 bp) without CPG1 or CPG3 indicated that CPG1 and CPG3 might be involved in transcriptional regulation of T1R1 gene. Subsequently, high DNA methylation decreased expression of T1R1 in intestinal tract. It could be a new mechanism to explain, at least partially, the vegetarian adaptation of grass carp by regulation of expression of umami receptor via epigenetic modification.

## Introduction

Taste receptors belong to G-protein coupled receptors, which were firstly cloned and characterized in topographically distinct subpopulations of taste receptor cells and taste buds^1^. Then they were identified in non-gustatory tissue including the gut, pancreas, and even the brain, suggesting their functional roles in other tissue^2^. T1R1/T1R3 and T1R2/T1R3 heteromers are activated by amino acids and sugars, respectively, which are preferable tastants. T1R1 is pseudogenized or absent in western clawed frogs^3^; fructivorous, insectivorous and vampiric bats^4^; giant pandas^5,6^ and marine carnivorous pinnipeds^7,8^. The pseudogenization of T1Rs in giant panda has been hypothesized to be related to its dietary switch from carnivore to herbivore, which is confirmed by the approximate match in inferred time between T1R1 pseudogenization and dietary switch of panda^6^. The pseudogenization of T1R1 in pinnipeds might be consistent with its feeding behavior of swallowing food whole without mastication and its carnivorous food habit^8^. The absence or presence of intact T1R1 are concordant with food habit formation, suggesting the adaption of T1R1 evolution to food habit transition and environmental changes. Previous studies on closely related species with high dietary diversity could provide a better understanding of the relationship between food habits and the evolution of umami taste^9^. However, little is known about the relationship between the presence/absence of intact T1Rs and food habits in fish.

The food habits of fish, which have higher sensitivity to water-soluble chemicals than mammals^10^, are highly species-specific and associated with chemosensory-mediated locomotion, especially taste sense^11^. The T1R1 gene has been reported to be lost in herbivorous blunt snout bream (*Megalobrama amblycephala*) by whole-genome analysis^12^. However, little information is known about the function and evolution of umami taste receptor gene nor the relationship with food habits^13^. The grass carp (*Ctenopharyngodon idella*) is an ecologically appealing model of vertebrate herbivores and goes through a transition from carnivore to herbivore during its life cycle. When its total length is shorter than 3 cm, the grass carp is carnivorous and feeds on zooplankton or benthos; fish 3–5.5 cm in length undergo food transition stages from zooplankton or benthos to aquatic macrophytes; when the fish grow to larger than 5.5 cm, they are entirely herbivorous^14–16^.

In addition to pseudogenization, the sensitivity of T1R1/T1R3 to umami compounds, which could be affected by genetic factors such as SNP variation and epigenetic modifications, might also be related to the food habits. In humans, some nonsynonymous single polymorphisms (nsSNP) in the coding region of T1R1 and T1R3 were demonstrated to be associated with the inability to taste monosodium glutamate in non-tasters and hypotasters^17,18^. In mice, saccharin preference is associated with nsSNPs in T1R3 gene^19^. However, little has been reported about the function of DNA methylation in the gene regulatory region of T1R1 and T1R3. DNA methylation modification plays crucial roles in the regulation of gene expression, such as modulating the initiation of gene transcription by the methylation of CpG islands in the gene control region^20,21^.

In the present study, the evolution of umami receptor T1R1/T1R3 in fish with different food habits was analyzed. We detected the responses of T1R1 and T1R3 to amino acids in grass carp. In addition, we investigated the DNA methylation profiles and mRNA expression of T1R1 and T1R3 genes in grass carp before and after the transition from carnivore to herbivore. These results might provide new insight into the function and evolution of umami taste receptors during food habit formation in fish.

## Results

### Sequence analysis of T1R1 and T1R3 genes in grass carp

**Figure 1:**
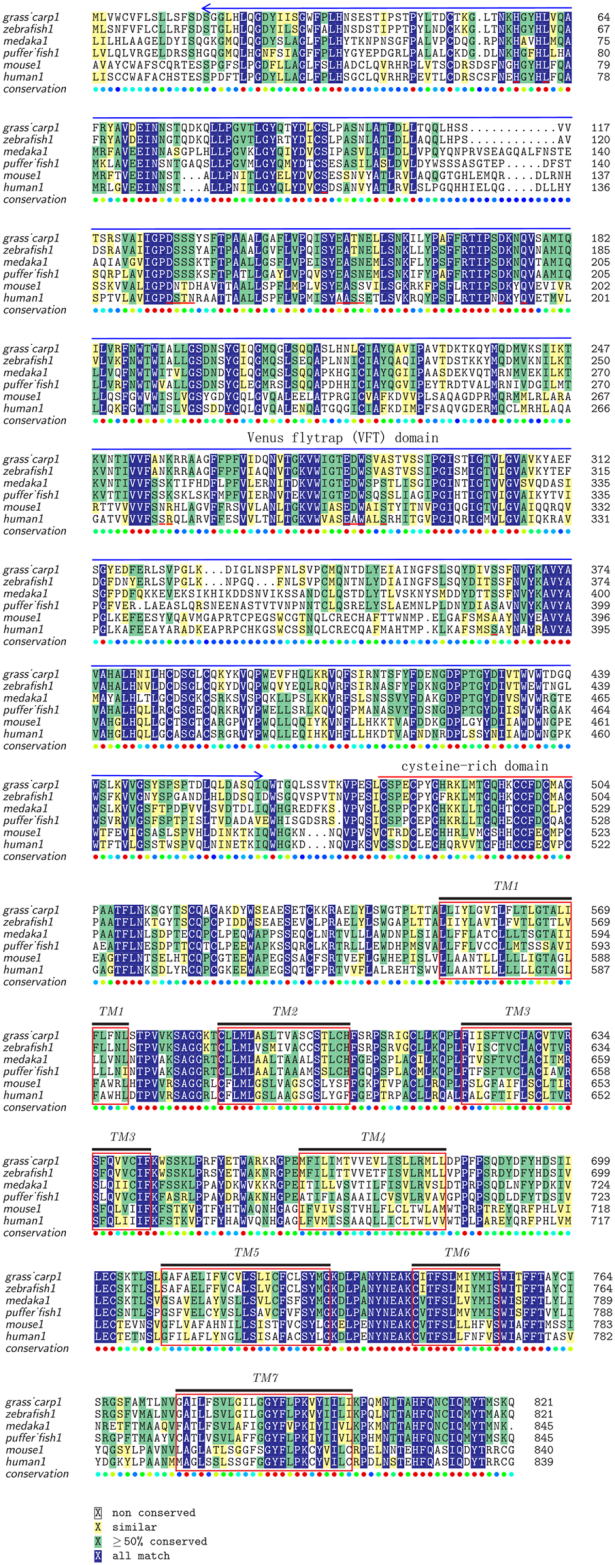
Multiple amino acid sequence alignment of T1R1. The colors indicate that the amino acids in that column were identical across all species. A long extracellular amino-terminal domain called a Venus flytrap module (VFTM) containing the ligand binding pocket was marked with blue line. A cysteine-rich domain (CRD), which links the VFT and 7TM domains was marked with red line. Transmembrane domains (TMD) of grass carp T1R1 were marked with red boxes. Amino acid sequences are available from the DDBJ/EMBL/GenBank databases shown in Table 3.

**Figure 2:**
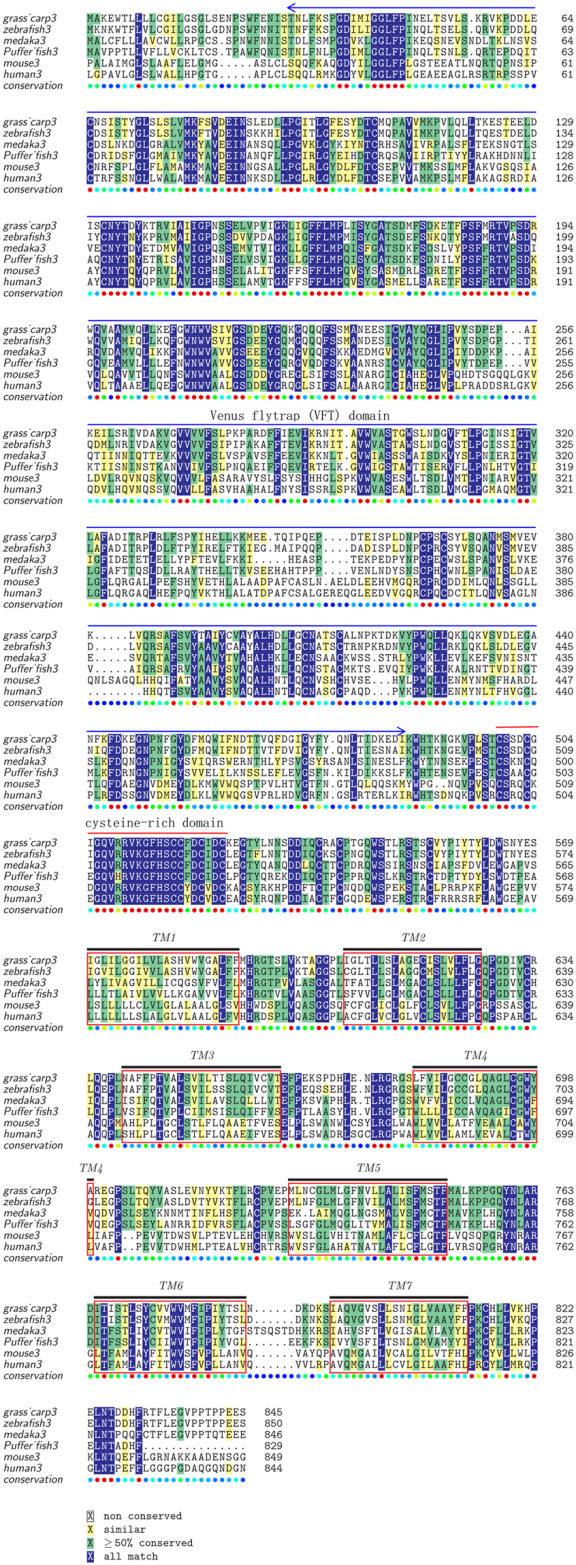
Multiple amino acid sequence alignment of T1R3. The colors indicate that the amino acids in that column were identical across all species. Extracellular amino-terminal domain Venus flytrap module (VFTM) containing the ligand binding pocket was marked with blue line. A cysteine-rich domain (CRD), which links the VFT and 7TM domains was marked with red line. Transmembrane domains (TMD) of grass carp T1R3 were marked with red box. Amino acid sequences are available from the DDBJ/EMBL/GenBank databases shown in Table 3.

**Figure 3:**
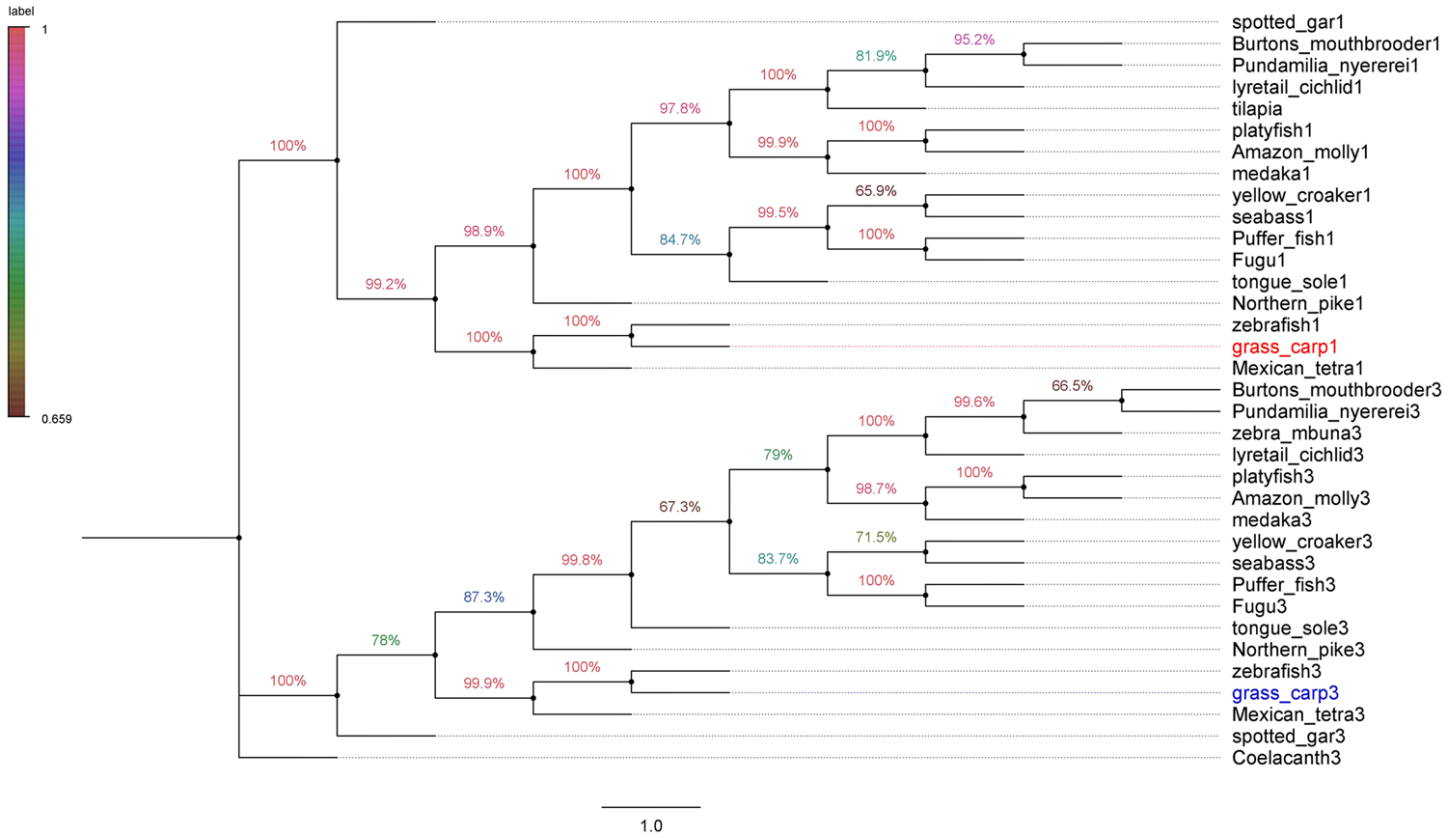
Phylogenetic analysis of fish T1R1 and T1R3. A phylogenetic tree was generated by the Maximum Likelihood method of MEGA7.0 (http://www.megasoftware.net/). The numbers at branch points represent bootstrap values (1000 repetitions). The scale bar indicates the relative measure of evolutionary distance. Amino acid sequences available in the EMBL/GenBank databases and food habit information of fish are shown in Table 3.

The complete CDS of grass carp T1R1 and T1R3 genes was 2466 bp and 2538 bp in length, encoding a 822-aa protein and a 845-aa protein, respectively (Gene Bank: KU976428, KU976429). As shown in Tables 1 and 2, the multiple alignments revealed the high conservation of amino acid sequences of T1R1 and T1R3 genes. The predicted structure of T1R1 and T1R3 in grass carp exhibited the characteristics of G protein coupled receptors, including a ligand-binding domain, a cysteine-rich domain at the extracellular N-terminal, and seven transmembrane regions (Fig. 1 and 2). No pseudogenization or frameshift mutations of T1R1 and T1R3 were found in fish with different food habits (Table 3). Phylogenetic analysis suggested that T1R1 and T1R3 of grass carp were most evolutionarily related to zebrafish, rather than a product of independent evolution for its herbivorous food habits (Fig. 3).

**Table 1:** Amino acid sequence identities of T1R1 compared with other fish and mammals.

	grass carp1	zebrafish1	Mexican tetra1	spotted gar1	tongue sole1	seabass1	puffer fish1	fugu1	platyfish1	medaka1	human1	mouse1
grass carp1	100.00											
zebrafish1	85.21	100.00										
Mexican tetra1	67.32	66.02	100.00									
spotted gar1	58.56	57.09	56.45	100.00								
tongue sole1	60.86	58.36	60.82	55.95	100.00							
seabass1	59.26	56.77	59.44	54.76	72.19	100.00						
puffer fish1	57.60	56.00	58.41	52.50	70.07	70.34	100.00					
fugu1	60.29	58.21	58.17	53.93	69.95	72.28	85.09	100.00				
platyfish1	59.90	57.91	60.20	56.90	71.43	71.53	69.31	71.24	100.00			
medaka1	59.54	57.67	61.05	56.02	70.45	70.81	65.59	67.86	79.11	100.00		
human1	41.31	40.10	43.35	43.42	41.58	40.56	41.38	41.25	40.92	42.26	100.00	
mouse1	41.18	40.67	44.03	43.13	41.09	39.83	40.12	39.76	40.80	41.54	73.37	100.00

Amino acid sequences available in the EMBL/GenBank databases and food habit information of fish are shown in Table 3.

**Table 2:** Amino acid sequence identities of T1R3 compared with other fish and mammals.

	grass carp3	zebrafish3	Mexican tetra3	spotted gar3	tongue sole3	seabass3	puffer fish3	Fugu3	platyfish3	medaka3	human3	mouse3
grass_carp3	100.00											
zebrafish3	83.91	100.00										
Mexican_tetra3	66.63	64.73	100.00									
spotted_gar3	55.87	54.69	57.85	100.00								
tongue_sole3	54.69	53.73	53.82	52.63	100.00							
seabass3	57.81	55.66	58.11	54.92	67.74	100.00						
puffer_fish3	54.18	52.00	55.68	54.59	63.74	70.82	100.00					
Fugu3	53.33	51.55	55.44	53.67	63.50	70.82	81.79	100.00				
platyfish3	52.91	51.78	52.54	51.72	61.76	67.49	59.78	61.41	100.00			
medaka3	52.33	51.14	53.69	51.43	59.64	65.12	59.20	58.72	65.85	100.00		
human3	37.61	36.88	37.94	39.95	34.99	37.13	38.56	37.08	35.09	36.31	100.00	
mouse3	36.03	35.07	36.01	38.04	34.34	36.08	36.60	35.60	34.93	35.42	73.38	100.00

Amino acid sequences available in the EMBL/GenBank databases and food habit information of fish are shown in Table 3.

**Table 3:** Food habit information for the fish studied.

Species	Dietary habits	EMBL/GenBank databases
coelacanth (*Latimeria chalumnae*)	Carnivore	T1R3: XP005986165.1
spotted gar (*Lepisosteus oculatus*)	Carnivore	T1R1: XP006642104.1 T1R3: XP006642093.1
tongue sole (*Cynoglossus semilaevis*)	Carnivore	T1R1: XP008316256.1 T1R3: XP008317882.1
northern pike (*Esox lucius*)	Carnivore	T1R1: XP010874113.1 T1R3: XP010893894.1
Burton’s mouthbrooder (*Haplochromis burtoni*)	Carnivore	T1R1: XP005941260.1 T1R3: XP005943730.1
lyretail cichlid (*Neolamprologus brichardi*)	Carnivore	T1R1: XP006785823.1 T1R3: XP006783296.1
Pundamilia nyererei (*Pundamilia nyererei*)	Carnivore	T1R1:XP005733129.1 T1R3: XP005724813.1
puffer fish (*Tetraodon nigroviridis*)	Omnivore	T1R1: CAG03219.1
fugu (*Takifugu rubripes*)	Omnivore	T1R1: BAE78486.1 T1R3: BAE78489.1
zebrafish (*Danio rerio*)	Omnivore	T1R1: NP001034614.2T1R3: NP001034717.1
grass carp (*Ctenopharyngodon idella*)	Herbivore	T1R1: KU976428 T1R3: KU976429
Mexican tetra (*Astyanax mexicanus*)	Carnivore	T1R1: XP007246390.1 T1R3: XP007251586.1
Amazon molly (*Poecilia formosa*)	Omnivore	T1R1: XP007553500.1 T1R3: XP007549797.1
medaka (*Oryzias latipes*)	Omnivore	T1R1: BAE78481.2 T1R3: AB200909.1
platyfish (*Xiphophorus maculatus*)	Omnivore	T1R1: XP005800206.1 T1R3: XP005816500.1
tilapia (*Oreochromis niloticus*)	Omnivore	T1R1: XP013126540.1
yellow croaker (*Larimichthys crocea*)	Carnivore	T1R1: KKF08786.1 T1R3: XP010729654.1
European seabass (*Dicentrarchus labrax*)	Carnivore	T1R1: DLAgn00132940,T1R3: DLAgn00094770

Sources of information: http://animaldiversity.ummz.umich.edu/.

### Gene structure of T1R1 and T1R3 genes in grass carp

**Figure 4:**
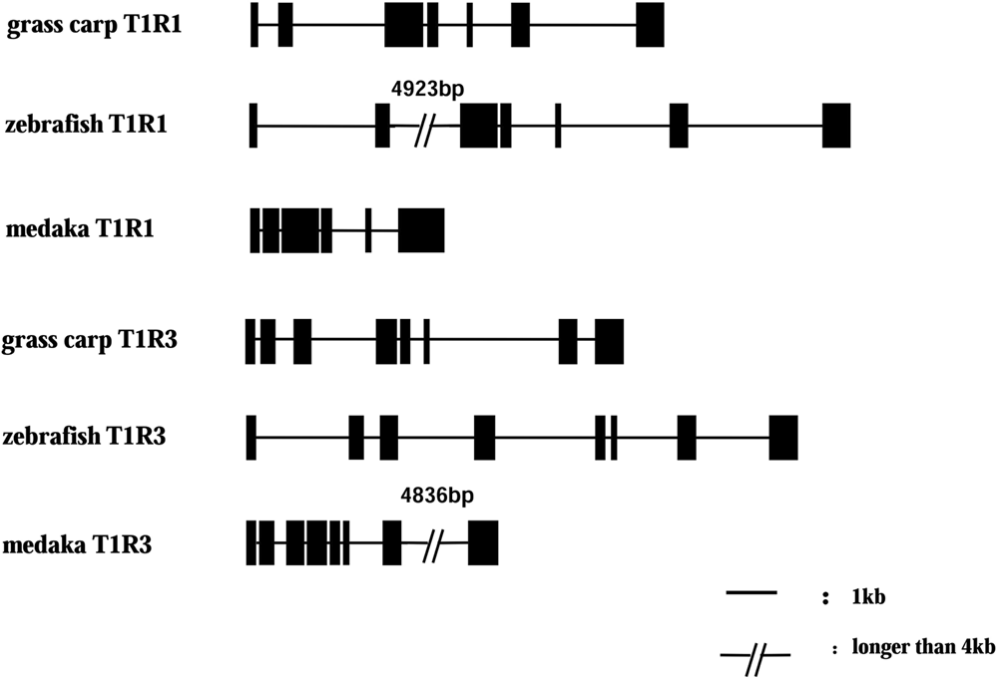
The gene structures of T1R1 and T1R3 genes in grass carp, zebrafish and medaka. The black lines indicate introns and the black boxes indicate exons. Grass carp and zebrafish T1R1 genes contain 7 exons and 6 introns. However, medaka T1R1 has only 6 exons and 5 introns, and the last intron is lost compared with grass carp and zebrafish. Grass carp, zebrafish and medaka T1R3 genes all contain 8 exons and 7 introns.

The total size of T1R1 gene we obtained from grass carp was 8150 bp in length, and contained 7 exons and 6 introns. The T1R3 gene of grass carp was 7425 bp in length, and contained 8 exons and 7 introns (Fig. 4).

### Synteny analysis of T1R1 and T1R3 genes in fish

**Figure 5:**
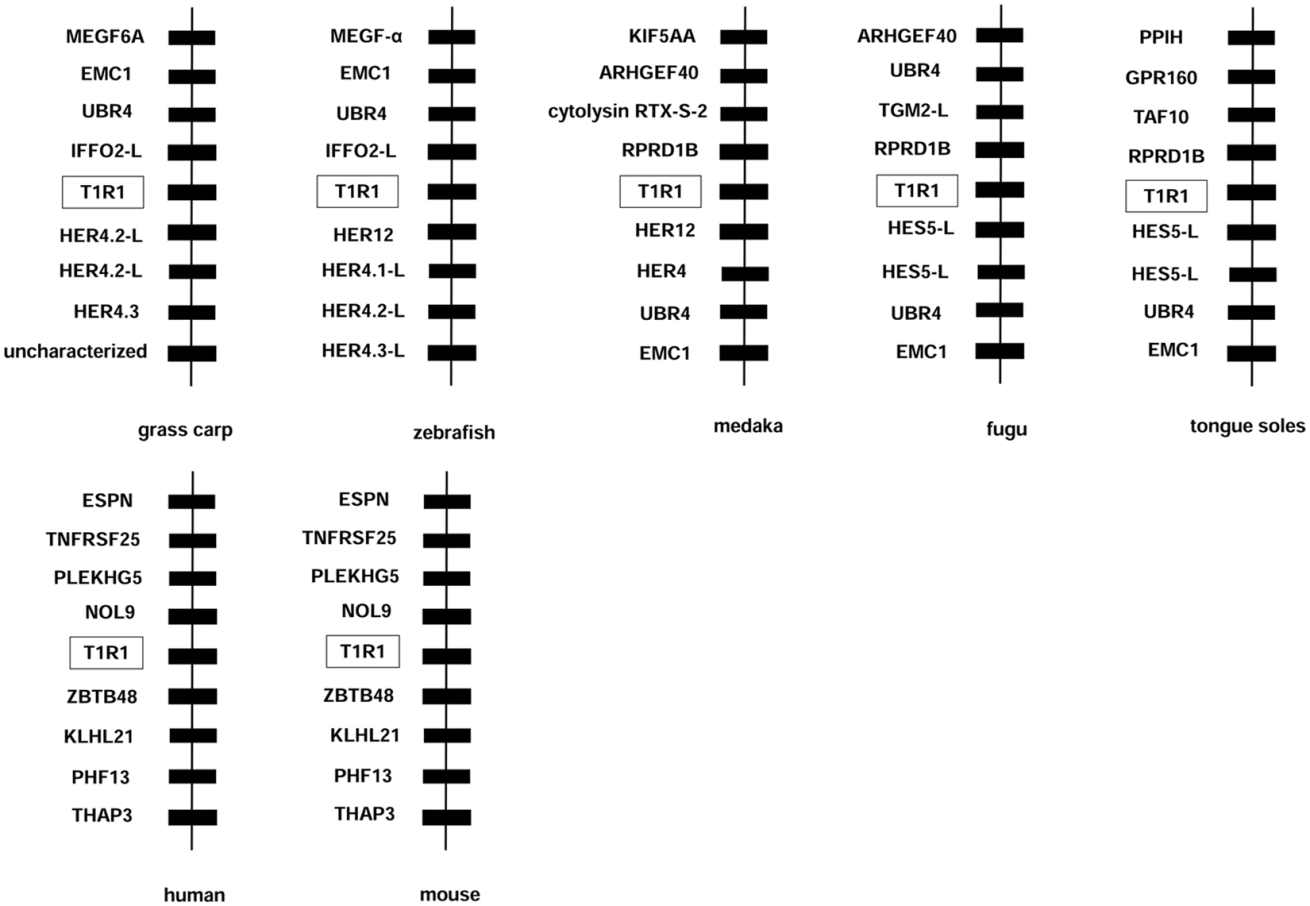
Synteny analyses of T1R1 genes. Adjacent genes of human and mouse T1R1 were strictly conserved. In fish transcription factor genes (hairy-related genes: HER4.1, HER4.2, HER4.3, HER12, hes family bHLH transcription factor 5: HES5) were near T1R1. ER membrane protein complex subunit 1 (EMC1), ubiquitin protein ligase E3 component n-recognin 4 (UBR4) appeared in downstream of T1R1 in medaka, fugu and tongue sole; however, it was located upstream of T1R1 in zebrafish and grass carp. Upstream genes of T1R1 in grass carp were similar as in zebrafish. The regulation of nuclear pre-mRNA domain containing 1B (RPRD1B) was present upstream of T1R1 in medaka, fugu and tongue sole but not in zebrafish and grass carp.

Synteny analysis showed that the genes adjacent to T1R1 and T1R3 genes were strictly conserved in humans and mice. In fish (Fig. 5), transcription factor genes (hairy-related genes: HER4.1, HER4.2, HER4.3, HER12, hes family bHLH transcription factor 5: HES5) were nearby T1R1. ER membrane protein complex subunit 1 (EMC1) and ubiquitin protein ligase E3 component n-recognin 4 (UBR4) appeared on the downstream of T1R1 in medaka, fugu and tongue sole; however, it was located on the upstream of T1R1 in zebrafish and grass carp. The upstream genes of T1R1 in grass carp were similar to those in zebrafish. Regulation of nuclear pre-mRNA domain containing 1B (RPRD1B) gene was present on the upstream of T1R1 in medaka, fugu and tongue sole, but not in zebrafish and grass carp.

**Figure 6:**
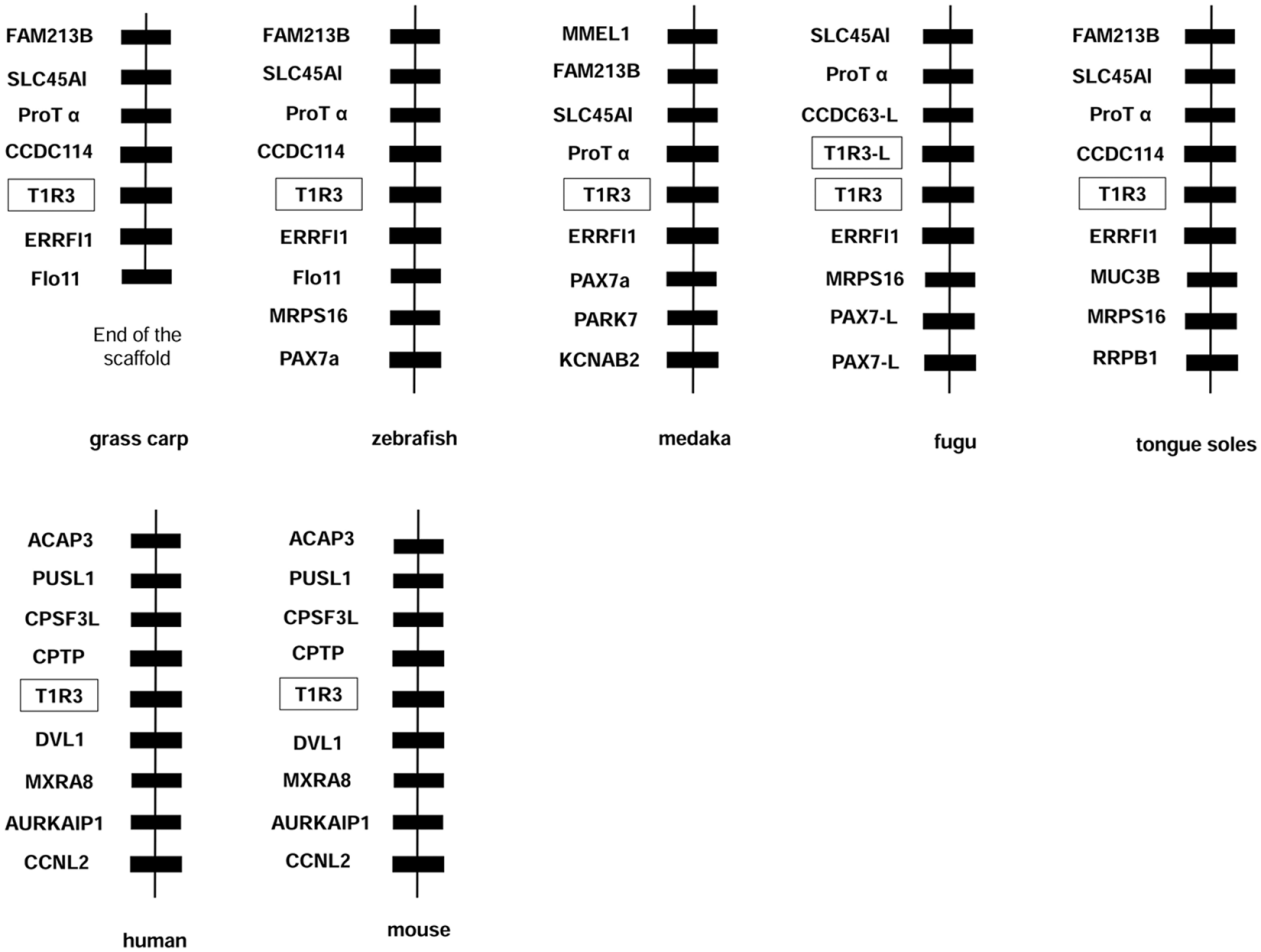
Synteny analyses of T1R3 genes. The adjacent genes of T1R3 were partially conserved. The solute carrier family 45 member 1 (SLC45A1), prothymosin alpha-like (ProT α), coiled-coil domain containing 114 (CCDC114) genes located upstream of T1R3 in most fishes. The ERBB receptor feedback inhibitor 1 (ERRFI1) was always next to the T1R3 gene in fish. Mitochondrial ribosomal protein S16 (MRPS16) and paired box 7a (PAX7a) genes are frequently downstream of fish T1R3.

In the fish we analyzed, the adjacent genes of T1R3 were partially conserved. The solute carrier family 45 member 1 (SLC45A1), prothymosin alpha-like (ProT α), and coiled-coil domain containing 114 (CCDC114) genes were located on the upstream of T1R3 in most fishes. The ERBB receptor feedback inhibitor 1 (ERRFI1) gene was always next to T1R3 gene in fish, and the mitochondrial ribosomal protein S16 (MRPS16) and paired box 7a (PAX7a) genes were frequently on the downstream of T1R3 in fish (Fig. 6).

### Response of grass carp T1R1 and T1R3 to L-amino acids

**Figure 7:**
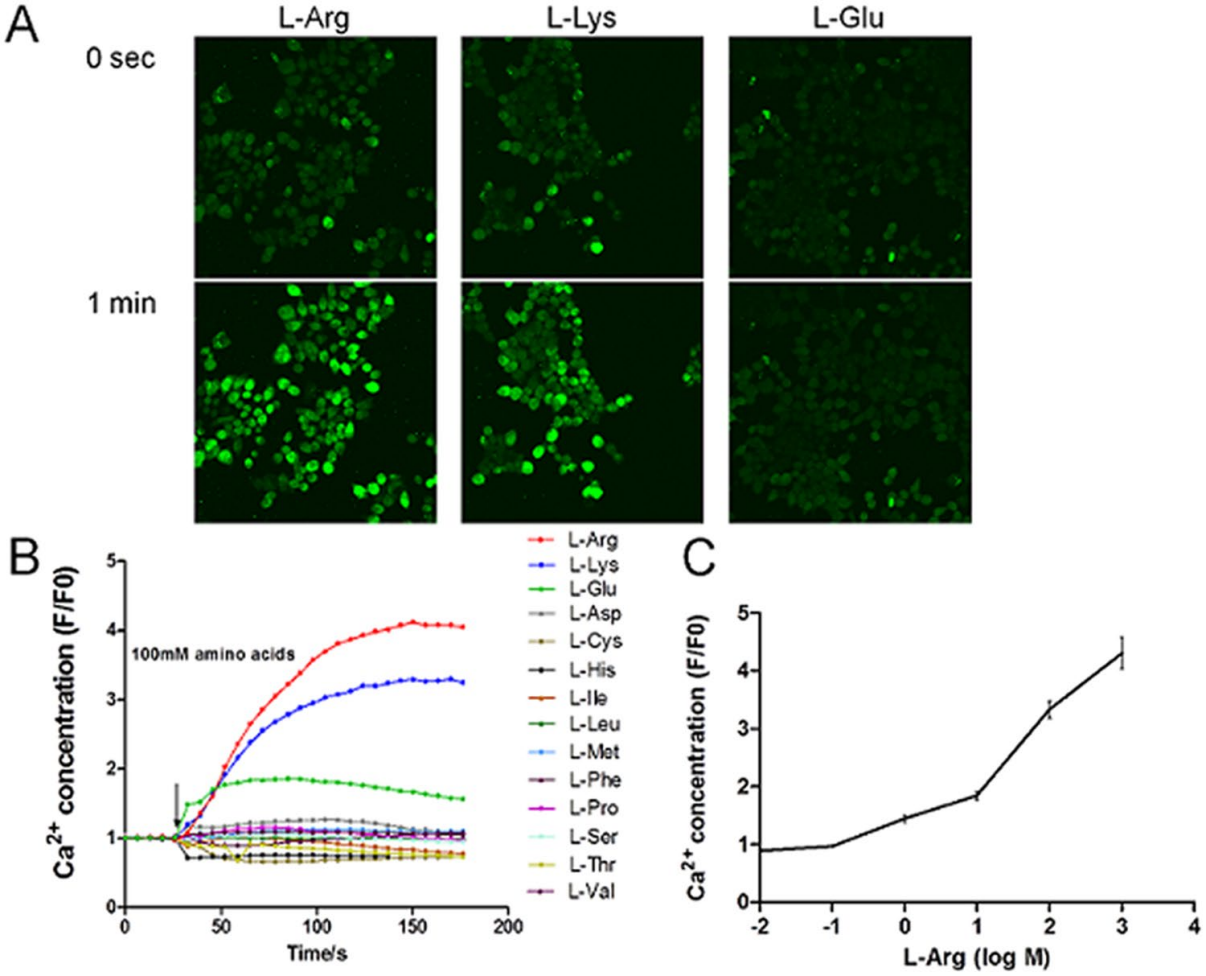
Responses of HEK293T cells co-expressing grass carp T1R1 and T1R3 to tastant stimuli. (**A**) Representative radiometric images of fluo-4AM-loaded HEK293T cells during the application of 100 mM L-Arg, L-Lys and L-glu. Top and bottom panels show the cell images that were obtained 0 s and 1 min after stimulation, respectively. (**B**) The quantification of responses of grass carp T1R1- and T1R3-transfected cells. Amino acids were used at 100 mM. (**C**) Dose-dependent responses of grass carp T1R1 and T1R3 to L-Arg. Responses were normalized to the mean response before tastant stimuli.

The cells with co-expression of grass carp T1R1 and T1R3, strongly responded to L-Arg and L-Lys, moderately to L-Glu and weakly to the other amino acids (Fig. 7A,B). The cell response to L-Arg were dose-dependent in the range of 0.01 mM to 1000 mM (Fig. 7C).

### mRNA expression of T1R1 and T1R3 in grass carp before and after food habit transition

**Figure 8:**
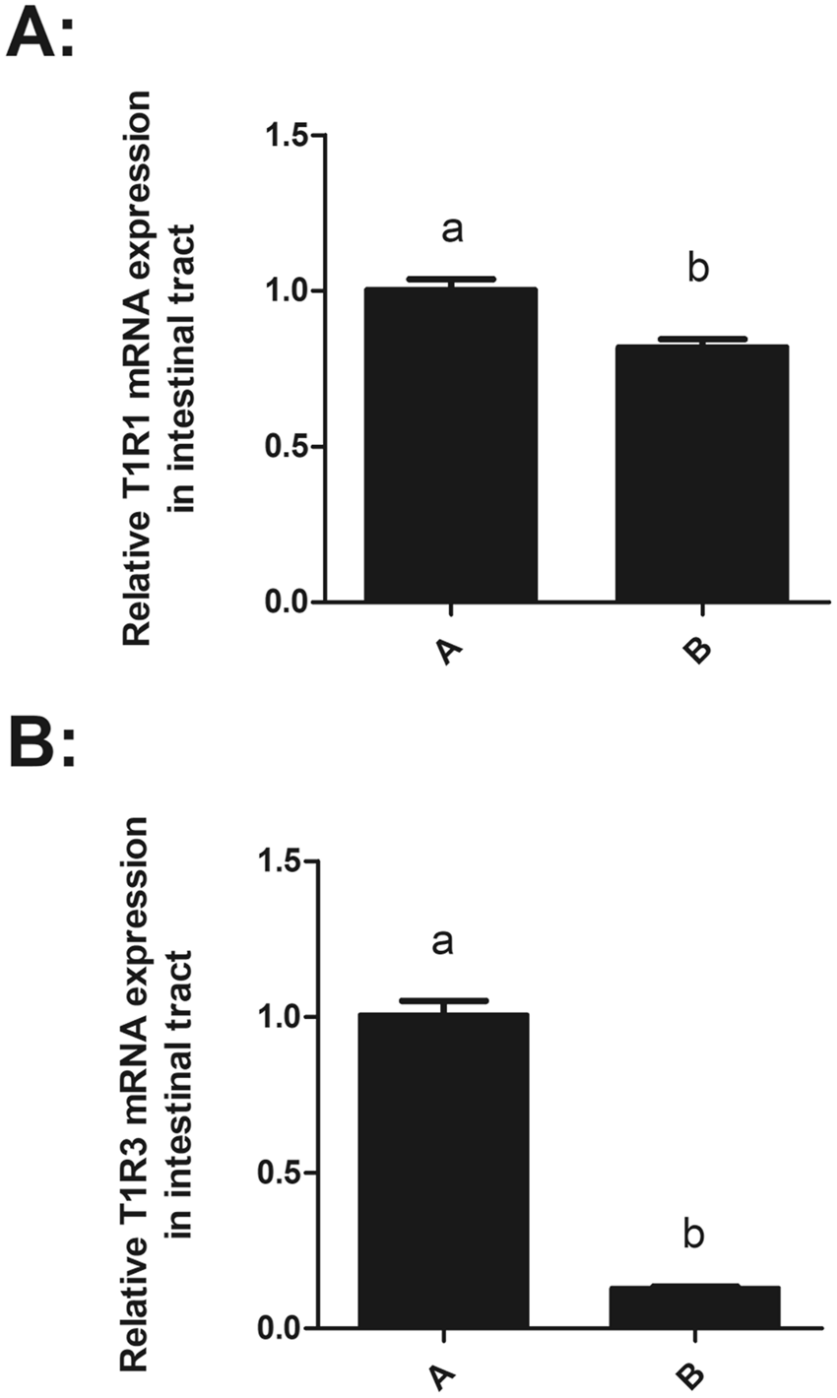
The T1R1 and T1R3 mRNA expression levels of the intestinal tract in the grass carp transition from carnivory to herbivory. (**A**) mRNA expression levels of T1R1 gene in the intestinal tract of grass carp transition from carnivory to herbivory. (**B**) mRNA expression levels of T1R1 gene in the intestinal tract of grass carp transition from carnivory to herbivory. The T1R1 and T1R3 gene expression levels were both significantly reduced in the grass carp brain and intestinal tract after the food habit transition to herbivory. All values represented the mean ± S.E.M. (n = 6).

As shown in Fig. 8, compared with fish before food habit transition, the gene expression of T1R1 and T1R3 were significantly reduced in the intestinal tract of grass carp after the food habit transition from carnivore to herbivore.

### Methylation of T1R1 and T1R3 in grass carp before and after the food habit transition

**Figure 9:**
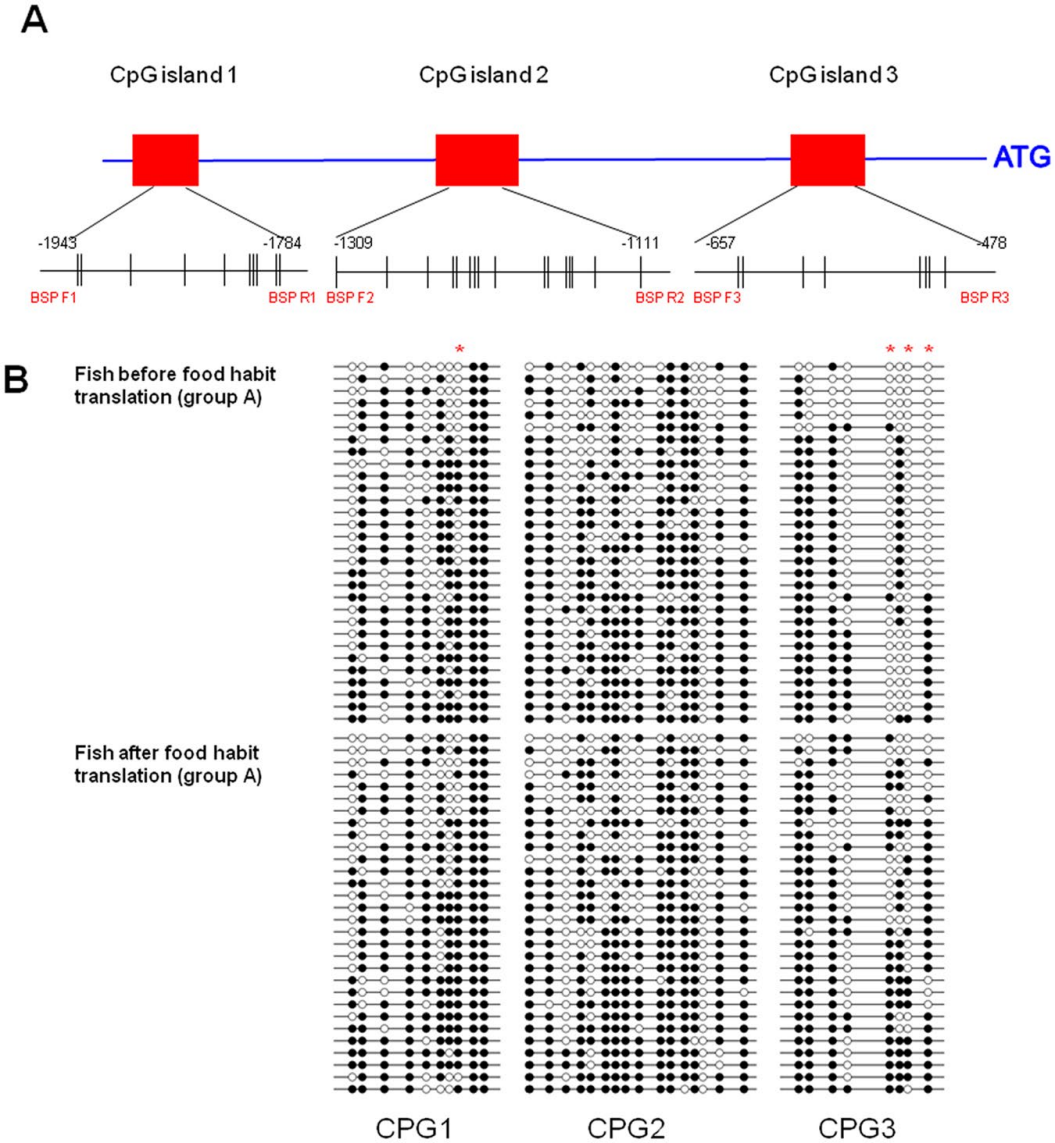
Differentially expressed CpG islands and DNA methylation patterns of T1R1 CpG islands in the grass carp intestinal tract. (**A**) Illustration of the region of CpG islands and BSP primers. The BSP detection region contained 3 CpG islands, which included 10 CpG sites, 16 CpG sites and 8 CpG sites, respectively. (**B**) DNA methylation patterns of the intestinal tract in grass carp before or after the food habit transition to herbivores as analyzed by BSP. Each line represents one individual bacterial clone, and each circle represents one single CpG dinucleotide. Open circles show unmethylated CpGs and black circles show methylated CpGs. A red asterisk suggests that the DNA methylation of the CpGs was significantly different after the food habit transition from carnivore to herbivore in grass carp.

We analyzed the CpG islands at −2000 bp upstream from the transcription initiation site (designated as 0) of T1R1 and T1R3 by methylation analysis software. As shown in Fig. 9, three CpG islands existed in this region of grass carp T1R1 gene. The first was −1943 to −1784 bp, the second was −1309 to −1111 bp and the last was −657 to −478 bp of T1R1 gene. The first CpG island contained 10 CpG sites, and the DNA methylation levels at the −1827 site was significantly higher in the intestine of grass carp after the food habit transition than those before the transition (Table 4). The second CpG island contained 16 CpG sites, and no difference in the DNA methylation levels was found in the intestine of grass carp before and after the food habit transition (Table 5). The last CpG island contained 8 CpG sites, and DNA methylation levels at the sites of −529, −522 and −500 CpGs were significantly higher in intestine of the grass carp after food habit transition (Table 6). However, no CpG island in this region was predicted in T1R3 of grass carp (not shown).

**Table 4:** Methylation status of each CpG in CpG island 1 of the T1R1 gene in grass carp before or after the food habit transition.

CpG position	−1921	−1916	−1891	−1861	−1845	−1833	−1830	−1827	−1813	−1807	Total
Me-CpG Fish before food habit transition (Group A)	11/30 36.3%	25/30 83.3%	21/30 70.0%	23/30 76.7%	13/30 43.3%	19/30 63.3%	20/30 66.7%	16/30 53%	30/30 100.0%	30/30 100.0%	204/298 68.5%
Me-CpG Fish after food habit transition (Group B)	12/30 40.0%	19/29 65.5%	21/30 70.0%	26/30 86.7%	17/29 56.7%	21/30 70.0%	15/30 50.0%	24/30 80.0%	30/30 100.0%	30/30 100.0%	214/298 71.8%
significance	—	—	—	—	—	—	—	*	—	—	—

*P < 0.05 ^—^*P* > 0.05.

**Table 5:** Methylation status of each CpG in CpG island 2 of the T1R1 gene in grass carp before or after the food habit transition.

CpG position	−1309	−1288	−1272	−1259	−1254	−1241	−1236	−1231	−1220	−1196	−1191	−1179	−1174	−1172	−1156	−1127	Total
Me-CpG Fish before food habit transition (Group A)	27/30 90.0%	27/30 90.0%	3/30 10.0%	20/30 66.7%	20/30 66.7%	12/30 40.0%	26/30 86.7%	14/30 46.7%	16/30 53.3%	24/30 80.0%	24/30 80.0%	24/30 80.0%	25/30 83.3%	0/30 0.0%	21/30 70.0%	28/30 93.3%	311/480 64.8%
Me-CpG Fish after food habit transition (Group B)	26/30 86.7%	21/30 70.0%	4/30 13.3%	25/30 83.3%	15/30 50.0%	18/30 60.0%	27/30 90.0%	15/30 50.0%	17/30 56.7%	26/30 86.7%	28/30 93.3%	27/30 90.0%	23/30 76.7%	0/30 0.0%	24/30 80.0%	24/30 80.0%	320/480 66.7%
significance	—	—	—	—	—	—	—	—	—	—	—	—	—	—	—	—	—

*P < 0.05 ^—^*P* > 0.05.

**Table 6:** Methylation status of each CpG in CpG island 3 of the T1R1 gene in grass carp before or after the food habit transition.

CpG position	−633	−627	−600	−587	−529	−524	−522	−500	Total
Me-CpG Fish before food habit transition (Group A)	28/30 93.3%	24/30 80.0%	25/30 83.3%	9/30 30.0%	2/30 6.7%	16/30 53.3%	1/30 3.3%	11/30 36.7%	116/240 48.3%
Me-CpG Fish after food habit transition (Group B)	28/30 93.3%	26/30 86.7%	25/30 83.3%	8/30 26.7%	21/30 70.0%	19/30 63.3%	11/30 36.7%	22/30 73.3%	160/240 66.7%
significance	—	—	—	—	*	—	*	*	—

*P < 0.05 ^—^*P* > 0.05.

### Luciferase activity assay of the upstream regulatory region of T1R1 in grass carp

**Figure 10:**
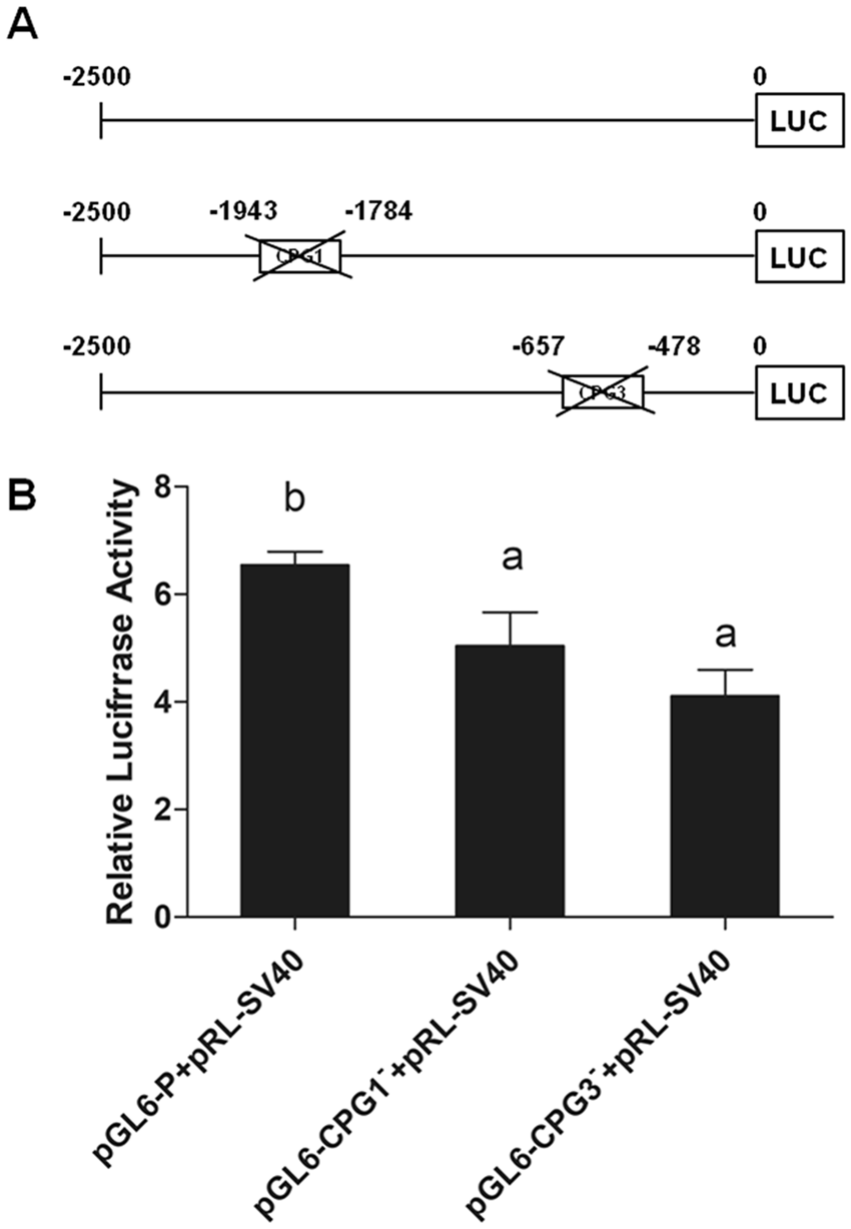
Relative activity of the upstream regulatory region of T1R1 gene. (**A**) Schematic representation of the upstream regulatory region of T1R1 gene. A series of plasmids containing the upstream sequences of grass carp T1R1 of different lengths (PGL6-T1R1-P, PGL6-T1R1-CPG1^−^, PGL6-T1R1-CPG3^−^) fused in the frame to the luciferase were co-transfected with pRL-SV40 into HEK293T cells. (**B**) Relative activity of the upstream regulatory region of T1R1 gene with deletions. Values were normalized to the control plasmid pGL6-Basic and represented as the ratio between firefly and Renilla luciferase activities. The results are expressed as the mean ± SEM arbitrary units of three independent experiments (*P* < 0.05).

Three recombinant vectors with the upstream sequences of grass carp T1R1 of different lengths (PGL6-T1R1-P, PGL6-T1R1-CPG1^−^, PGL6-T1R1-CPG3^−^) were successfully constructed (Fig. 10A). They were then co-transfected into HEK293T cells with plasmid pRL-SV40-N as the internal control, and the luciferase activity was detected at 36 h after transfection. Dual luciferase assay showed that compared with the cells with PGL6-T1R1-P plasmid, the luciferase activity of the cells with PGL6-T1R1-CPG1^−^ and PGL6-T1R1- CPG3^−^ plasmid were significantly decreased (Fig. 10B).

## Discussion

The pseudogenization or absence of T1R1 gene in western clawed frogs^3^, giant pandas^5,6^, pinnipeds^7,8^ and blunt snout bream has been hypothesized to be related to food habits. These studies have reported that the absence or presence of functional T1R1 was concordant with food habits, suggesting the adaptive evolution of T1R1 to food habit transition or environmental changes. However, this concordance between genetic and behavioral evidence has been challenged^9^. Research has suggested that additional factors shape T1R1 evolution, such as in the relatively closely related species, the high dietary diversity can impact on the evolution of T1R1 and umami taste^4^. In the present study, no pseudogenization or frameshift mutations of T1R1 and T1R3 genes were found in the herbivore grass carp. T1R1 and T1R3 of grass carp were most evolutionarily related to zebrafish, as they belong to Cyprinidae, rather than a product of independent evolution with its herbivorous food habit. It is suggested that the evolution of umami taste receptor genes in fish could be independent of feeding ecology.

T1R1 and T1R3 have been identified in several mammals^22–24^, zebrafish, medaka and puffer fish^25^. In the present study, we cloned the complete coding sequence of T1R1 and T1R3 genes in grass carp. The predicted secondary structures of T1R1 and T1R3 proteins were conserved not only in fish but also in mammals^23,25^. Synteny analysis revealed that the genes adjacent to T1R1 or T1R3 genes were strictly conserved in humans and mice, while no obvious synteny was found in fish, especially T1R1 gene, suggesting a more complicated evolutionary relationship in fish. Genes adjacent to T1R1 were more variable than those adjacent to T1R3, suggesting the potential unique transcription regulation and interactions of umami receptor genes with adjacent gene in fish. Interestingly, hairy-related genes and the hes family were conservatively located on the downstream of T1R1 gene in fish. Her factors have been demonstrated to play important roles in developmental and neurogenic processes, including somitogenesis and components of the central nervous system as well as the neuro-sensory system^26^. The conserved gene locus in fish might be attributed to the co-evolution of sensory system in fish.

Li *et al*.^23^ showed that T1R1/T1R3 heterodimers respond to AAs in mammals. Oike *et al*.^27^ revealed that unlike mammals, T1R2/T1R3 was not activated by sugars in medaka or zebrafish. Zebrafish mainly detect amino acids by T1R2s/T1R3 rather than by T1R1/T1R3. Medaka could detect amino acids by both T1R2/T1R3 and T1R1/T1R3. Vertebrate T1R2s have probably evolved to adapt themselves to necessary nutrients depending on the species. However, in the present study, the results revealed that T1R1/T1R3 of grass carp strongly responded to L-Arg and L-Lys, moderately to L-Glu and weakly to other amino acids. In addition, we identified more isoforms of T1R2 genes in grass carp than zebrafish and medaka. All T1R2s were responded to glucose or fructose *in vitro* (unpublished data). The results were different from those of zebrafish and medaka fish. Facial nerve recordings and calcium imaging using an HEK293T heterologous expression system in zebrafish demonstrated that the fish perceived to L-Ala and L-Pro but not to sugras, mainly by T1R2s/T1R3, Calcium imaging analysis of T1Rs medaka fish revealed that both T1R1/T1R3 and a series of T1R2/T1R3 responded to amino acids (strongly to L-Arg and L-Ser) but not to sugars^27^. Although the gene sequences of T1R1 and T1R3 were highly conserved in grass carp and zebrafish, the diversity of amino acids sequence in ligand-binding domain of T1R1 might raise the possibility of different binding affinity and response to amino acids between grass carp and zebrafish.

Previous studies have demonstrated that amino acid recognition in humans and mice could be determined by separate sets of amino acids in VFTD, which is the key domain for the sensitivity of umami taste receptor^27–29^. Raliou *et al*.^30^ reported that amino acid substitutions (A110V and R507Q) in the N-terminal ligand-binding domain of T1R1 in humans leads to the impaired response of T1R1/T1R3 to monosodium glutamate *in vitro*. The amino acid composition was different in aquatic macrophytes (main food source of grass carp) and tubificidae, daphnia, or the fish body (main food sources of zebrafish and medaka)^31,32^. We hypothesize that the different responses of T1R1 and T1R3 to amino acids might contribute to their preferences for plant protein or animal proteins. These findings might offer a good explanation for the high species specificity in amino acid preferences and food habits in teleosts^24,27^.

Research has revealed that the activation of T1R1/T1R3 initiated the peristaltic reflex and pellet propulsion in the distal colon^33^. In addition, the activation of T1R1/T1R3-initiated sensing of amino acids by the gut-expressed T1R1/T1R3 could stimulate CCK secretion in the proximal intestine of mice^34^, providing evidence of the significant role of T1R1/T1R3 in nutrient sensing in the intestinal tract. The presence of taste receptor mRNA has been reported in the midgut of rainbow trout^35^. Evidence of the existence of taste receptors in fish guts have suggested that the sensing of food might also have functional effects beyond oral taste sensations. In our previous research^16^, grass carp fed with duckweed had significantly greater growth than fish before the food habit transition or those without a food habit transition fed with chironomid larvae. It has been suggested that a longer gut could enable fish to achieve higher growth rates on plant materials, which are relatively poor in nutritional quality^16^. These results have indicated the importance of gastrointestinal development in the food habit transition of grass carp. For the study of evolution of the food habit transition from carnivory to herbivory in grass carp, we have focused on the umami receptors in the gut of grass carp. A comparative analysis between carnivores and herbivores in one species eliminated the differences in results from different species^15,16^.

The upstream regulatory region of T1R1 gene was methylated in grass carp both before and after the food habit transition. DNA methylation levels at the sites of −1827, −529, −522 and −500 in CpG1 and CPG3 were significantly higher in grass carp after the food habit transition than those before the transition. The transcription factor binding sites that are sensitive to methylation were predicted in these differentially methylated regions. We also found that the upstream regulatory region of T1R1 (−2500-0 bp) without CPG1 or CPG3 sequences showed lower transcriptional promotion than those with CPG1 or CPG3 by luciferase activity assay, indicating that the CPG1 and CPG3 might involved in the transcriptional regulation of T1R1 gene. However, no CPG island of T1R3 gene was found in grass carp although the expression results revealed a drastic transcriptional down-regulation after food habit transition, suggesting T1R3 might not be regulated through DNA methylation during food habit transition of grass carp. T1R3 is more crucial for taste perception and could also combine with T1R2 to respond to sweet substance. There could exist other mechanism for transcriptional regulation of T1R3 gene which was unidentified at present. The higher DNA methylation levels of the T1R1 gene were correlated with its lower expression in grass carp after the food habit transition than those before the food habit transition. Previous studies indicated that umami tastes were strictly dependent on T1R receptors, and the selective elimination of T1R subunits by knockout could differentially abolish behavioral and nerve responses to umami compounds^36,37^. As the qualitative changes in the amino acid profiles of the diets of grass carp before and after the food habit transition from carnivory to herbivory, the reduced expression levels of the umami receptor gene suggested an insensitivity to umami taste, which might be the cause of vegetarian adaptation in grass carp. It could be a new mechanism for the vegetarian adaptation of grass carp by the regulation of the expression of the umami receptor via DNA methylation of the umami receptor gene in addition to the pseudogenization or frameshift mutation of the umami receptor gene.

In conclusion, no pseudogenization or frameshift mutations of T1R1 and T1R3 were found in grass carp, which was different from other herbivorous fish, the blunt snout bream. The T1R1/T1R3 in grass carp strongly responded to L-Arg and L-Lys, moderately to L-Glu and weakly to other amino acids, which was different from the response in zebrafish and medaka. The gene expression of T1R1 was significantly decreased in grass carp after the food habit transition, which might be attributed to the higher DNA methylation levels in the upstream regulatory region of T1R1 with CPG islands. This might be a new adaptation mechanism for amino acid preferences and food habits in vertebrates.

## Materials and Methods

All animals and experiments were conducted in accordance with the “Guidelines for Experimental Animals” of the Ministry of Science and Technology (Beijing, China). The study was approved by the Institutional Animal Care and Use Ethics Committee of Huazhong Agricultural University. All efforts were made to minimize suffering.

### Cloning of grass carp T1R1 and T1R3 gene sequences

To obtain the T1R1 and T1R3 gene sequences in grass carp, we searched the genome database of grass carp available at the official National Center for Gene Research website (http://www.ncgr.ac.cn/grasscarp/). In the first step, TBLASTN searches were conducted with *E*-value 10^−10^ against the genomic data using the coding sequences (CDS) of T1R1 and T1R3 identified from the genome databases of zebrafish *Danio rerio*, medaka *Oryzias latipes* and fugu *Takifugu rubripes*^23^. The obtained sequences were confirmed by BLASTP searches against NCBI non-redundant (nr) database, if the best hit was a previously known T1R1 and T1R3, it was considered a candidate sequence. In the second step, each region of BLAST similarity was extended in 5′ and 3′ directions to establish a detailed prediction of the coding sequences. The exon-intron junctions were determined by comparing the genomic sequence with the cDNA sequence using SPIDEY (http://www.ncbi.nlm.nih.gov). In the third step, the cDNA of lip and tongue were used to verify the sequences. Polymerase chain reaction (PCR) was conducted on Biometra Thermo cyclers (Biometra, Germany) using Phanta^®^ Super-Fidelity DNA Polymerase (Vazyme Biotech, China) with the designed primers (Table 7).

**Table 7:** Nucleotide sequences of the primers.

Primers	Sequences (5′-3′)
***Primers for cloning of T1R1 and T1R3***	
CLgc T1R1 F	ATGTTGGTCTGGTGTGTGTTTCTCTC
CLgc T1R1 R	TCACTGCTTGGACATGGTGTACATT
CLgc T1R3 F	ATGGCTAAGGAGTGGACGCTT
CLgc T1R3 R	CTAGCTTTCTTCAGGTGGTGTTGG
***Primers for construction of expression vector***	
EXgc T1R1 F	TAGTCCAGTGTGGTGGAATTCATGTTGGTCTGGTGTGTGTTTCTCTC
EXgc T1R1 R	GGTTTAAACGGGCCCTCTAGATCACTGCTTGGACATGGTGTACATT
EXgc T1R3 F	TAGTCCAGTGTGGTGGAATTCATGGCTAAGGAGTGGACGCTT
EXgc T1R3 R	GGTTTAAACGGGCCCTCTAGA CTAGCTTTCTTCAGGTGGTGTTGG
***Primers for genomic DNA amplicon***	
CPG1 T1R1 F	TGTTTAAGTGCTATATCAGGTCCC
CPG1 T1R1 R	CAAAGGTCTTACGGCTGTGGAAT
CPG2 T1R1 F	GCCGCTCAGATATTGCTCCC
CPG2 T1R1 R	CATTTCTGTCAAACCCAACCTCTG
CPG3 T1R1 F	ACAGAGGTTGGGTTTGACAGAA
CPG3 T1R1 R	GTGTGAGCAAAAACACGCAGTT
***Primers for BSP amplicon***	
BSP1 T1R1 F	AGAAAATATGAAAAAGGATAATTT
BSP1 T1R1 R	ACTTTACCTCGCTTACGAAAAC
BSP2 T1R1 F	GAGAATATTTTTGGTGCGTTAAAA
BSP2 T1R1 R	ATATTATAAAACGACGAAAATATT
BSP3 T1R1 F	AGGTATATGTAGTCGATGAGTTGA
BSP3 T1R1 R	CCCTATTTAAATATAAACAAAAACAC
***Primers for cloning of the upstream regulatory region of T1R1 gene***	
gc T1R1-P F	GGTACCGCTTGGGTGTCGAGGTTGTG
gc T1R1-P R	CTCGAGGTTACTTCCTCTGATATGAGCTCTTC
gc T1R1-CPG1^−^A F	GGTACCGCTTGGGTGTCGAGGTTGTG
gc T1R1-CPG1^−^A R	CTTCAAAAATACTTACATTTAAGGCTTGCCAAAACAAAGTT
gc T1R1-CPG1^−^B F	ACTTTGTTTTGGCAAGCCTTAAATGTAAGTATTTTTGAAGGGTTA
gc T1R1-CPG1^−^B R	CTCGAGGTTACTTCCTCTGATATGAGCTCTTC
gc T1R1-CPG3^−^A F	GGTACCGCTTGGGTGTCGAGGTTGTG
gc T1R1-CPG3^−^A R	GTTTTTGTATGACCCTTTAAAGAAGTTACAACTTTTAGAATGCAT
gc T1R1-CPG3^−^B F	TTCTAAAAGTTGTAACTTCTTTAAAGGGTCATACAAAAACTGCGT
gc T1R1-CPG3^−^B R	CTCGAGGTTACTTCCTCTGATATGAGCTCTTC
***Primers for real-time PCR***	
RTgc EF1 F	GCTGACTGTGCCGTGCTGAT
RTgc EF1 R	GCTGACTTCCTTGGTGATTTCC
RTgc T1R1 F	CTGCCGCCACCTTCCTCA
RTgc T1R1 R	CTCCGGCCGACTTCACCAC
RTgc T1R3 F	CACAGGATGGTCTCTGAACG
RTgc T1R3 R	ATAGGGACTGAATAGGCGAA

### Sequence analysis of T1R1 and T1R3

The sequences of grass carp T1R1 and T1R3 genes were analyzed by the program Editseq to translate into amino acid sequences using standard genetic codes. Multiple alignments of amino acid sequences were performed by ClustalW2 (http://www.ebi.ac.uk/Tools/msa/clustalw2/). The phylogenetic tree based on the amino acid sequences was constructed by the Maximum Likelihood method of the MEGA 7.0 program (http://www.megasoftware.net/index.html) and decorated by iTOL (http://itol.embl.de/). Numbers at the branches reflect the confidence levels as obtained by bootstrapping (1000 replications).

To determine whether T1R1 and T1R3 genes of grass carp are orthologous to other vertebrate species, synteny analysis was performed by searching the flanking gene(s) of T1R1 and T1R3 in human, mouse, zebrafish, medaka, fugu (*Takifugu rubripes*) and tongue sole (*Cynoglossus semilaevis*) genomes using the Ensembl genome browser (http://www.ensembl.org/index.html), UCSC Genome Bioinformatics (http://genome.ucsc.edu/index.html) and NCBI map viewer (http://www.ncbi.nlm.nih.gov/mapview/).

### Grass carp T1R1/T1R3 co-expressing cells and Ca^2+^ imaging analysis

The complete coding sequences of T1R1 and T1R3 genes of grass carp were subcloned into the pcDNA3.1 expression vector (Invitrogen) using ClonExpress^TM^ II (Vazyme Biotech, China). Human embryonic kidney 293T (HEK293T) cells were cultured in Dulbecco’s modified Eagle’s medium (DMEM) (Cat. No. C11995500, Life Technologies, Carlsbad, CA, USA) supplemented with 10% fetal bovine serum (Sigma-Aldrich, Saint Louis, MO) at 37 °C in 10% CO_2_. After 18 hours, the cells were transiently transfected with grass carp T1R1 and T1R3 gene using Lipofectamine 2000 reagent (Invitrogen, Carlsbad, CA). Cells transfected with empty vector, lacking T1R1 or T1R3, were employed to control for unspecific reactions of the cellular background. Eighteen hours after transfection, the cells were washed with Hanks balanced salt solution (HBSS) (Sigma-Aldrich, Saint Louis, MO). Cells were loaded with 4 µM of the calcium indicator dye fluo-4 AM (Cat. No. F10489, Invitrogen, Carlsbad, CA) diluted in HBSS for 30–40 min at 37 °C and then rinsed and incubated in 1 ml of HBSS for >30 min. The stimulation was performed by the addition of 1 ml amino acid by pipette. Cells were transfected with the pEGFP-N1 vector, which can express green fluorescent protein, to evaluate the transfection efficiency. Thirty-six hours after transfection, the cells were washed with HBSS, and the green fluorescent protein was observed. The transfection efficiency was 70% to 80%.

The fluo-4 AM fluorescence intensities resulting from excitation at 494 nm were measured at 514 nm using an inverted confocal microscope (FluoView FV1000; Olympus, Tokyo, Japan). Baseline was established for at least 15 s before stimulation, as shown in the Fig. 7B. After that, cells were perfused with 100 mM L-amino acids (Sigma-Aldrich, Saint Louis, MO) at a rate of 2 ml/min. Images were recorded at 6.54-s intervals up to 183.16 s after the addition and analyzed using FV10-ASW 3.1 Viewer software. The backgrounds of the emission intensities were subtracted. Data are expressed as the ratio of the fluorescence intensities of several single 20 HEK293T cells per dish and initial intensity (F/F0).

### Fish and sample collection

Fish and samples were prepared according to the experiments of He *et al*.^16^. The grass carp embryos were obtained from Wuhan Academy of Agricultural Science and Technology (Wuhan, Hubei Province, China). Larvae were raised in tanks and fed with chironomid larvae *Chironomus tentans*. At 46 days post-hatch (dph) (body weight 0.39 ± 0.05 g, body length 28.05 ± 0.99 mm), 6 fish were randomly selected for sample collection as fish before food habit transition (Group A). The rest of the fish in Group B were fed with duckweed *Lemna minor* after the food habit transition to herbivores. Fish in both groups had free access to food 24 h a day and fed for 70 days. At 116 dph (body weight and body length for Group B were 7.34 ± 1.43 g and 72.78 ± 6.15 mm, respectively), 6 fish were randomly selected from the group for sample collection. The fish were deeply anesthetized with MS-222 (200 mg L^−1^) and killed by immediate spinal destruction for the measurement and dissection. The whole intestine of the grass carp before and after the food habit transition was collected and then frozen in liquid nitrogen and stored at −80 °C for RNA and DNA isolation. Total RNA was extracted using Trizol Reagent (TaKaRa, Japan), and then its integrity and quantity were measured using protein and agarose gel electrophoresis and an Eppendorf Biophotometer (Hamburg, Germany). According to the manufacturer’s instructions, cDNAs were obtained from 1 µg total RNA with the HiScript^®^ II Reverse Transcriptase (Vazyme Biotech, China) in a 20-μL reaction volume.

### DNA methylation analysis

Genomic DNA was extracted following the standard procedures using TIANamp Genomic DNA Kit (Tiangen, Beijing, China). DNA treatment with sodium bisulfite was performed using the EZ DNA Methylation Kit (Zymo Research, USA) according to the manufacturer’s protocol. The BSP primers were designed by the online MethPrimer software 14 and Primer 5.0 (Table 7). PCR was performed on Biometra Thermo cyclers (Biometra, Germany) by using Taq plus DNA Polymerase (Vazyme Biotech, China). The PCR products were gel purified using a Gel Purification Kit (Sangon, Shanghai, China) and then were subcloned into the pMD18-T clone vector (Takara, Japan). Ten positive clones for each subject were randomly selected for sequencing (Sangon, Shanghai, China). The final sequence results were processed by online QUMA software (http://quma.cdb.riken.jp/). Three samples from the intestine of grass carp before and after the food habit transition from carnivore to herbivore were analyzed.

### Luciferase activity assay of the upstream regulatory region of T1R1 in grass carp

The upstream regulatory region sequences of T1R1 in grass carp were cloned by overlapping PCR^38^ using Phanta^®^ Super-Fidelity DNA Polymerase (Vazyme Biotech, China), and specific primers containing Kpn I and Xho I restriction sites were designed (Table 7). As shown in Fig. 10A, three sequences were obtained, including the sequence of the upstream regulatory region of −2500 bp upstream from the transcription initiation site (designated as 0), the −2500 bp sequence without CPG1, the −2500 bp sequence without CPG3. For the generation of luciferase reporter, the PCR product was purified and subsequently digested using kpn I and Xho I restriction enzymes, then cloned into the corresponding restriction sites of pGL6-basic vector by a ClonExpress™ II One Step Cloning Kit (Vazyme, USA).

For determining the function of the upstream regulatory region of T1R1, transfection assays were performed. HEK293T cells were plated in 24-well plates for 24 h and then transfected with different plasmids with Lipofectamine 2000. Equimolar amounts (500 ng) of reporter plasmids were used in Opti-MEM (Gibco/Invitrogen), and they were co-transfected with 1 ng pRL-SV40, a Renilla luciferase reporter vector, as the internal control. After 4 h, the cell culture medium was replaced by 10% FBS-DMEM. Thirty-six hours later, transfected cells were washed twice with phosphate buffered saline (PBS) and then collected. The cells were lysed with Passive Lysis Buffer (Promega) and assayed for Firefly and Renilla luciferase activities in a luminometer by the Dual-Luciferase Reporter Assay System (Promega). The luciferase value of each sample was first normalized against the pRL-SV40 levels, and the relative light unit (RLU) intensity was presented as the ratio of firefly luciferase to Renilla luciferase. The results were normalized to the control reporter pGL6-Basic. All experiments were performed in triplicate and repeated three times.

### T1R1 and T1R3 gene expression analysis of the food habit transition from carnivore to herbivore in grass carp

To detect the expressions of T1R1 and T1R3 mRNA in grass carp before and after food habit transition from carnivore to herbivore, real-time PCR assays were carried out by quantitative thermal cycler (MyiQ™ 2 Two-Color Real-Time PCR Detection System, BIO-RAD, USA). Primers were designed according to the sequences we cloned (Table 7). The EF1 gene was used to normalize the template amount as an endogenous reference. All amplifications for each RNA sample were performed in triplicate. The reaction volume was 10 μL GoTaq^®^ qPCR Master Mix (BIO-RAD, USA), 1 μl of cDNA, and 0.2 μM of each primer. The PCR cycling parameters were 95 °C for 30 s followed by 40 cycles at 95 °C for 10 s, 58 °C for 30 sec and a melt curve step from 65 °C, gradually increasing from 0.5 °C·s^−1^ to 95 °C, with acquisition data every 6 sec. Gene expression levels were quantified relative to the expression of EF1 gene using the optimized comparative Ct (2^−ΔΔCt^) value method. The specificity of the primers was determined through sequencing and the melting curve of PCR products. Plasmid containing target fragments were dilated 10-fold. qPCR was conducted using different dilutions as templates to construct standard curves for genes. The amplification efficiencies were analyzed according to the slope of the standard curve in a given run.

### Statistical analysis

The normality of data was assessed by using SPSS 19.0 software with the Shapiro-Wilk test. All data of T1R1 and T1R3 gene expression levels were subjected to one-way analysis of variance using SPSS 19.0 software. Differences between the means were tested by Duncan’s multiple range test after the homogeneity of variances was checked. The DNA methylation analysis was determined with the χ^2^ test. Statistical significance was determined at the 5% level. All data were presented as the mean ± S.E.M (standard error of the mean).
